# Recent Progress in Contact Engineering of Field-Effect Transistor Based on Two-Dimensional Materials

**DOI:** 10.3390/nano12213845

**Published:** 2022-10-31

**Authors:** Jialei Miao, Xiaowei Zhang, Ye Tian, Yuda Zhao

**Affiliations:** 1 Department of Electrical Engineering and Computer Science, Ningbo University, Ningbo 315211, China; 2 School of Micro-Nano Electronics, Hangzhou Global Scientific and Technological Innovation Centre, Zhejiang University, 38 Zheda Road, Hangzhou 310027, China; 3 Key Laboratory of Optoelectronic Chemical Materials and Devices of Ministry of Education, Jianghan University, Wuhan 430056, China

**Keywords:** contact resistance, two-dimensional (2D) materials, Fermi-level pinning, transistor

## Abstract

Two-dimensional (2D) semiconductors have been considered as promising candidates to fabricate ultimately scaled field-effect transistors (FETs), due to the atomically thin thickness and high carrier mobility. However, the performance of FETs based on 2D semiconductors has been limited by extrinsic factors, including high contact resistance, strong interfacial scattering, and unintentional doping. Among these challenges, contact resistance is a dominant issue, and important progress has been made in recent years. In this review, the Schottky–Mott model is introduced to show the ideal Schottky barrier, and we further discuss the contribution of the Fermi-level pinning effect to the high contact resistance in 2D semiconductor devices. In 2D FETs, Fermi-level pinning is attributed to the high-energy metal deposition process, which would damage the lattice of atomically thin 2D semiconductors and induce the pinning of the metal Fermi level. Then, two contact structures and the strategies to fabricate low-contact-resistance short-channel 2D FETs are introduced. Finally, our review provides practical guidelines for the realization of high-performance 2D-semiconductors-based FETs with low contact resistance and discusses the outlook of this field.

## 1. Introduction

The development of silicon complementary metal–oxide semiconductor (CMOS) integrated circuits has followed Moore’s law for several decades. With the downscaling of the transistor dimensions into sub−20 nanometer nodes, two main challenges emerge, including drain-induced barrier lowering and degradation of the carrier mobility of Si. The former effect makes the transistor hard to turn off and results in a high off-current, which will cause high static power consumption. Emerging technologies such as FinFETs and Gate-All-Around (GAA) FETs have been employed to decrease the off-current. The mobility degradation is caused by the strong interfacial scattering, especially when the semiconductor thickness (t_b_) is in the sub−3−nm regime [1]. Since the discovery of graphene, two-dimensional (2D) materials with atomic thickness exhibit high carrier mobility, even at t_b_ < 3 nm [1], and show high immunity to the short-channel effect. These unique properties contribute to making 2D materials promising candidates to fabricate ultimately scaled transistors.

Although 2D materials present a unique pathway to build next-generation electronic devices, the construction of 2D-materials-based transistors (2D FETs) faces several technical challenges, including the growth of high-quality wafer-scale 2D materials [2], wafer-scale transfer methods [3], low contact resistance [4], and the high-quality dielectric interface [5]. These technical issues lead to large device-to-device variation [6] and the apparent discrepancy between the theoretical prediction and actual device performance, limiting the industrial applications of 2D materials in logic devices. Among these challenges, the contact problems are of vital important because the working mechanism of 2D FETs is based on the control of charge injection at the metal/2D junction, which is quite different from silicon CMOS transistors [4]. Recent studies show tremendous advances in the achievement of the ideal Mott–Schottky contact and the lowering of the contact resistance in 2D FETs [7,8,9]. The value of the contact resistance in 2D-materials-based transistors is approaching the requirement of the International Roadmap for Devices and Systems (IRDS) 2024 targets of logic transistors [10]. It is believed that a summary of emerging strategies to realize contact engineering in 2D FETs is urgently needed.

In this review, we present a comprehensive analysis of contact challenges in 2D FETs and discuss the recent research progress. We start with the origins of high contact resistance in 2D FETs. Then, two contact structures are presented, including top contact and edge contact, followed by several strategies to decrease the contact resistance. Finally, an outlook is provided to present the possible roadmap for the contact engineering of 2D FETs.

## 2. Fermi-Level Pinning

### 2.1. Fermi-Level Pinning and Pinning Factor

**Figure 1:**
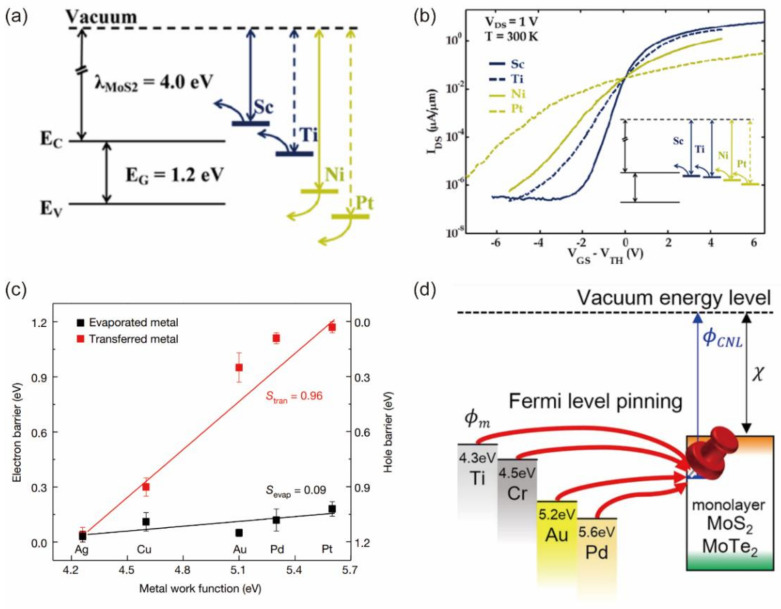
Origin and influence of Fermi-level pinning. (**a**) Expected metal Fermi level with the electronic bands of MoS_2_ and different metal electrodes with different work function. (**b**) Transfer curves with different metals. The inset shows the actual line-up based on the experimental data. Reprinted/adapted with permission from Ref. [26]. Copyright 2012, American Chemical Society. (**c**) Experimentally determined Schottky barrier height for transfer of different transfer metals and evaporated metals. Reprinted/adapted with permission from Ref. [13]. Copyright 2018, Springer Nature. (**d**) Schematic illustration of Fermi-level pinning. Reprinted/adapted with permission from Ref. [27]. Copyright 2017, American Chemical Society.

The Schottky barrier height ($\Phi_{\mathrm{SB}}$) and the contact resistance (*R*_c_) are important quantitative parameters to examine the quality of the metal–2D material junction. In an ideal metal–semiconductor junction, $\Phi_{\mathrm{SB}}$ is determined by the Schottky–Mott rule based on the energy-level band alignment [11,12]:

(1) $$\Phi_{\mathrm{SB},n}=\Phi_{M}-\chi_{s}\;$$

(2) $$\Phi_{\mathrm{SB},p}=I_{s}-\Phi_{M}\;$$

where $\Phi_{\mathrm{SB},n}$ and $\Phi_{\mathrm{SB},p}$ are the Schottky barrier heights for electrons and holes transport, $\Phi_{M}$ represents the metal work function, $\chi_{s}$ represents the electron affinity and $I_{s}$ represents the ionization potential of the semiconductor. The $\Phi_{\mathrm{SB}}$ is linearly dependent on the metal work function in the Schottky–Mott model, as shown in Figure 1a. However, the metal work function in FETs is always derived from the theoretical value, and it is pinned on a specific position within the bandgap of the semiconductor regardless of the selection of different metals, as shown in Figure 1b. This effect is called Fermi-level pinning, which makes the metal–semiconductor junction insensitive to the modulation of the metal work function. The pinning factor S represents the strength of Fermi-level pinning:

(3) $$S=|d\Phi_{\mathrm{SB}}/d\Phi_{M}|\;$$

The value of S in an ideal device is nearly equal to 1, but S is usually far away from 1 in 2D FETs. Liu et al. and Kim et al. demonstrated the S value of ~0.1 in MoS_2_ FETs with deposited metal contact [13,14]. The Fermi-level pinning effect strongly limits the performance of 2D semiconductor FETs.

### 2.2. Origins of Fermi-Level Pinning

Tersoff et al. successfully established a parameter-free metal-induced gap states (MIGS) model in bulk semiconductors to explain the Fermi-level pinning effect. The MIGS model can quantitatively explain the almost unchanged Schottky barrier height, which is independent of the metal work function in experiments [15]. Guo et al. employed the density functional theory (DFT) calculation to calculate the Schottky barrier height of 2D transition metal dichalcogenides (TMDs) by using different metal contacts. The calculated pinning factor is around 0.3, demonstrating a strong Fermi-level pinning effect. They found that direct bonding existed between the contact metal atoms and the chalcogen atoms of TMDs, leading to the MIGS [16]. The charge neutrality level serves as a quantitative characteristic of the electronic states of the defective semiconductor surface. Dominik et al. employed the primary theoretical model to calculate the charge neutrality level (CNL) of the monolayer TMDs, and they found that CNL is mostly placed near the mid-point of the semiconducting band gaps [17]. Although they use different theoretical calculation methods, both studies demonstrate that the MIGS can well-explain the Fermi-level pinning effect in 2D TMDs. Fermi-level pinning in 2D FETs mainly originates from the interfacial states. In the early stage of the study, Au, Ni and Pt with a high melting temperature are selected as the metal contact of 2D FETs, and they are deposited by evaporation or sputtering techniques, as shown in Figure 1a,b. This leads to the compact stacking of metal atoms on the surface of 2D layers, the wavefunction interaction between the metal and 2D semiconductor, and the rehybridizations of the semiconductor’s original wavefunctions, resulting in the strong orbital overlap and MIGS. Photolithography and electron-beam lithography are common techniques to pattern the electrodes on 2D materials. Compared with bulk semiconductors, atomically thin 2D materials are sensitive to laser [18], electron-beam [19,20] and chemical solution [21]. Matsunage et al. reported that a relatively low electron-beam dose (280 μC/cm^2^) used in conventional electron-beam lithography will induce strain in MoS_2_, leading to the local widening of the MoS_2_ bandgap [22]. Preeti et al. systemically reported the doping effect of the conventional lithography process and the used chemical solvent. For example, acetone shows n-type doping and chloroform displays p-type doping on MoS_2_ [23]. Meanwhile, the high-energy deposition process generates atomic defects at the interface of metal and 2D layers, facilitates the formation of covalent bonds [24,25], and gives rise to MIGS. Liu et al. experimentally showed that the typical metal deposition process induced defects at the contact region, which were observed by transmission electron microscope [13]. In 2D FETs with deposited metal contact, the injected charges are accumulated at the interfacial gap states regardless of the modulation of the metal work function, as shown in Figure 1c. The Fermi level is pinned around these gap states and a Schottky barrier is unavoidable, as shown in Figure 1d. Furthermore, heavy doping via ion implantation is employed in Si CMOS FETs to realize Fermi-level depinning and successfully achieve low contact resistance. However, the ion implantation cannot be well implemented on 2D materials. The implantation process will generate a large number of defects in atomically thin 2D materials and degrade the carrier transport.

Overall, the interface defects of the 2D semiconductor can induce the MIGS and greatly affect the contact quality. Furthermore, the conventional Fermi-level depinning methods in Si CMOS FETs are not suitable for 2D FETs. Therefore, the specific contact strategies should be tailored to meet the requirement of 2D FETs.

## 3. Contact Engineering of 2D FETs

FETs based on 2D materials can realize the ultimate downscaling of transistor dimensions. Along with the scaling of channel length, the scaling of contact region will produce new challenges. The most straightforward effect is the increase in contact resistance in the scaled contact region. The transfer length is the effective length with which carriers are transferred from the contact metal to channels. According to the current crowding model [28], carriers prefer to be transferred inside the more conductive metal and enter the semiconductor only near the end of metal–semiconductor contact regions, leading to a much smaller transfer length in comparison with the contact length. In this condition, the contact resistance is dependent on the transfer length, rather than the contact length, and its value can be calculated by the distributed resistor network model [29]. When the contact length is equal to or smaller than the transfer length, the charge injection is limited by the contact length. Therefore, a high-quality metal–semiconductor contact becomes more important in 2D FETs for ultra-scaled integrated circuits.

In order to overcome the strong Fermi-level pinning effect in 2D FETs, great progress has been made in the optimized metal-deposition process and the efficient carrier-modulation methods. In this section, state-of-the-art contact engineering strategies are systematically discussed.

### 3.1. Top Contact Engineering

Top contact refers to the deposition of metal contact on the surface of 2D layers. Due to the large surface-to-edge ratio, top contact is widely used in 2D FETs, and the fabrication process is simple and efficient. In this part, we introduce the use of bulk metals, bulk semimetals and 2D metals as top contact to optimize the contact of 2D FETs.

#### 3.1.1. Bulk Metals

**Figure 2:**
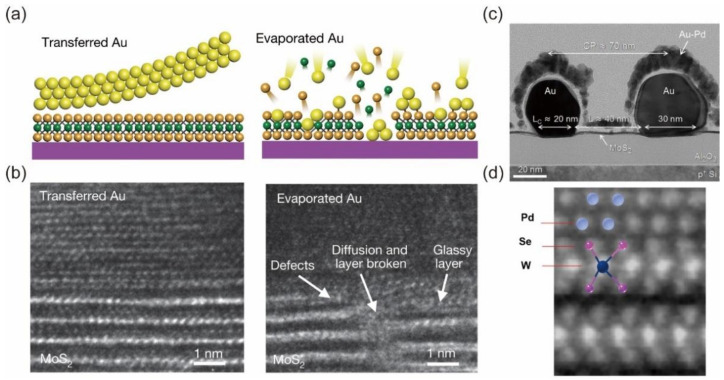
Atomical clean interface is significant to Fermi-level depinning. (**a**) cross-sectional schematics of transferred Au electrodes with atomically sharp and clean metal–semiconductor interfaces and conventional electron beam-deposited Au electrodes with poor interface. (**b**) Cross-section TEM images of transferred Au electrodes and evaporated Au electrodes. Reprinted/adapted with permission from Ref. [13]. Copyright 2018, Springer Nature. (**c**) TEM cross-section of a MoS_2_ FET with gold electrodes deposited under ultra-high vacuum. Reproduced with permission from Ref. [35]. Copyright 2016, American Chemical Society. (**d**) cross-sectional STEM of the Pd–WSe_2_ interface. Reprinted/adapted with permission from Ref. [8]. Copyright 2022, Springer Nature.

Defects in 2D materials, including intrinsic defects and the generated defect during the fabrication process, are the main origins of the Fermi-level pinning effect. Defects contribute to the interfacial trap states, which is directly related to the Fermi-level pinning effect. In early studies, bulk metals with low work function were used to achieve high-performance n-type 2D FETs. Kwon et al. reported the use of Al as a contact metal in MoS_2_ FETs and obtained a Schottky barrier height of 70 meV [30]. Das et al. reported the use of Sc as a contact metal in MoS_2_ FETs [26] due to the low work function of Sc (3.5 eV). An average Schottky barrier of 0.03 eV was achieved, which represents a very low contact barrier at that time. However, from the Schottky–Mott rule, the ideal Schottky barrier between Sc and MoS_2_ should be negative because the work function of Sc is much lower than the CBM of MoS_2_. The positive Schottky barrier experimentally demonstrates Fermi-level pinning. In order to avoid the generation of defects during the metal-deposition process, transferred metal methods have been developed to preserve a sharp and clean interface between metal and 2D layers. Liu et al. reported realization of the van der Waals (vdW) contact through mechanically transferring metals to avoid chemical bonding and defect-induced gap states, as shown in Figure 2a,b. Owing to the atomically clean interface between metal and semiconductor, the majority carrier type and corresponding Schottky barrier height are strongly dependent on the metal work function (S=0.96) [13]. Wang et al. further reported that the transferred metal can achieve low contact resistance in p-type WSe_2_ FETs [31]. Due to the successful Fermi-level depinning, the work function of the transferred metal plays an important role in determining the charge injection and the device performance. Han et al. reported the use of molecular functionalization to change the work function of gold electrodes. Then, they fabricated top-contact FETs via the transfer of these pre-modified electrodes to tune the charge injection in MoS_2_ FETs [32], demonstrating the modulation of the Schottky barrier. This method has also been used in 2D-materials-based resistive random-access memory, leading to a stable resistive switching performance [33]. Therefore, the transferred metal method represents a reliable way to create vdW contact [34], but wafer-scale metal transfer technology is still lacking. Moreover, the metal transfer process is mostly operated under the optical microscope and the alignment error is still huge, which is another big challenge.

The conventional metal-deposition process can be optimized to realize low contact resistance. Chris et al. reported that Au deposited in ultra-high vacuum (~10^−9^ Torr) yields three times lower contact resistance than that in normal conditions, as shown in Figure 2c [35]. Wang et al. reported high performance p-type FETs based on single− and few−layer MoS_2_ and WSe_2_ by the electron-beam evaporation of high-work-function metals such as Pd and Pt, as shown in Figure 2d [8]. They conducted the metal evaporation at a high vacuum (<10^−8^ torr) and low temperature (18 °C to 36 °C) to avoid high temperature damage to 2D semiconductors and to form vdW contact between the metal and semiconductor interface. Wang et al. reported the employment of In metal to build van der Waals contact with 2D semiconductors [36]. Due to the low melting point of In, the temperature of the 2D sample can be greatly decreased and a high-quality vdW interface can form, resulting in the Fermi-level depinning. Furthermore, In metal can be used to form stable alloys with other metals to modulate the work function. The use of In alloy as contact shows the advantages in the preservation of a high-quality contact interface and the effective tunability of the Schottky barrier. Kumar et al. employed In/Au alloy and Sn/Au alloy as contact electrodes in monolayer MoS_2_ FETs, and they achieved an ultra-low contact resistance of 190 Ω·μm for In/Au alloy and 270 Ω·μm for Sn/Au alloy [37]. The use of metal alloys increases the thermal stability of low-melting-point metals and results in ~450 °C temperature tolerance that is compatible with back-end-of-line (BEOL).

#### 3.1.2. Bulk Semimetals

**Figure 3:**
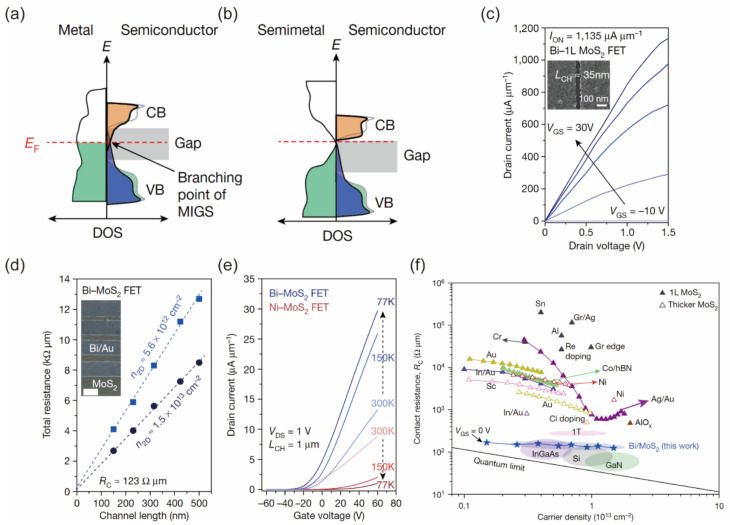
Band structure of normal metal contact (**a**) and bismuth–semiconductor contact (**b**). The Fermi level of the semi-metal aligns with the conduction band of the semiconductor, the density of states at the Fermi level of the semimetal is near-zero, and metal-induced gap states are suppressed. (**c**) Output curves of a 35-nm Bi−MoS_2_ FET with a high current density. (**d**) Contact resistance of MoS_2_ FETs with bismuth electrodes. (**e**) Transfer curves of Bi−MoS_2_ and Ni−MoS_2_ FETs at various temperatures. (**f**) State-of-the-art contact technology for MoS_2_ transistors as a function of $n_{2d}$. Reprinted/adapted with permission from Ref. [7]. Copyright 2021, Springer Nature.

Recently, a powerful strategy was demonstrated by adopting semi-metals as contact electrodes to suppress MIGS and the Fermi-level pinning effect [7,38]. Shen et al. used semi-metal Bi as the n-type contact metal in monolayer MoS_2_ FETs [7]. Bi as a semi-metal has a negligible density of state at the Fermi level, and this induces the suppression of MIGS, as shown in Figure 3a,b. Furthermore, the use of Bi contact results in the degenerately doped MoS_2_ with a high electron density of 1.5 × 10^13^ cm^−2^, and the Fermi level shifts from inside the bandgap to above the conduction band minimum. They achieved an ultralow contact resistance of 123 Ω·μm and a high current density of 1135 μA μm^−1^ in a 35−nm channel length MoS_2_ FETs, as shown in Figure 3c,d. Owing to the highly efficient carrier injection between Bi and MoS_2_, the drain current density increased by lowering the temperature from room temperature to 77 K, as shown in Figure 3e. The values of Bi−MoS_2_ contact resistance are comparable to those Si transistors and approach the quantum limit, as shown in Figure 3f. However, it has been observed that the MoS_2_ FETs with Bi electrodes degraded severely after annealing at 300 and 400 °C [39]. Chou et al. reported semimetal antimony (Sb) as a novel contact metal to enable 2D materials towards advanced electronic device applications. They obtained a near-zero Schottky barrier height and a low contact resistance of 0.66 kΩ·μm [39]. Compared with Bi contact electrodes, the melting point of Sb (630 °C) is much larger than that of Bi (271 °C), although Sb has a higher work function than Bi. The transfer curves of MoS_2_ FETs with Sb electrodes show a better electrical performance after high-temperature annealing. Overall, the use of semi-metal as a contact can greatly reduce MIGS and realize Fermi-level depinning in 2D FETs.

#### 3.1.3. Two-Dimensional Metals/Semimetals

**Figure 4:**
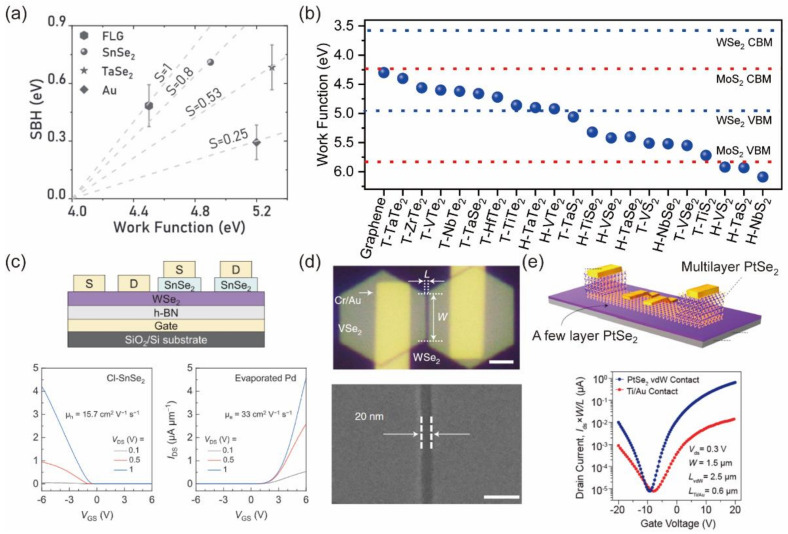
(**a**) Fermi-level de-pinning with vdW contacts. Reprinted/adapted with permission from Ref. [41]. Copyright 2021, John Wiley and Sons. (**b**) Band alignment of MoS_2_ and WSe_2_ with 2D metals and semimetals [42]. (**c**) Schematic and transfer curves of WSe_2_ devices contacted with both evaporated Pd and Cl–SnSe_2_ electrodes. Reprinted/adapted with permission from Ref. [45]. Copyright 2022, John Wiley and Sons. (**d**) Optical microscopy and SEM images of the bi-layer WSe_2_ transistor. Reprinted/adapted with permission from Ref. [9]. Copyright 2022, Springer Nature. (**e**) Schematic illustration of few-layer PtSe_2_ FETs with metallic PtSe_2_ vdW contact and a conventional Ti/Au contact. Reprinted/adapted with permission from Ref. [50]. Copyright 2021, American Chemical Society.

The MIGS are commonly found at the interface between 3D metal and 2D semiconductors. Liu et al. theoretically found that the interface states in the metal–semiconductor junction mainly derive from the 3D metal rather than the 2D semiconductor [40]. Therefore, they suggested replacing the 3D bulk metal with 2D metals. In their works, they predict that the Fermi-level pinning effect can be greatly suppressed when the 2D metal–2D semiconductor interface is well formed. The existence of a van der Waals distance between the 2D metal and the 2D semiconductor (3 to 4 Å) leads to the weak interlayer interaction, mild orbital overlap and the creation of interface dipole, contributing to Fermi-level depinning. Two-dimensional layered materials with metallic properties, such as graphene, 1T−MoS_2_ and PtSe_2_, can be used to form vdW contacts on 2D semiconductors. Majumdar et al. employed 2H−TaSe_2_, graphene and degenerately-doped semiconducting SnSe_2_ as contact metals [41]. They demonstrated that vdW contacts exhibited a universal Fermi-level depinning phenomenon, as shown in Figure 4a.

We present the theoretical band alignment of MoS_2_ and WSe_2_ with different 2D metals/semimetals, as shown in Figure 4b [42]. The stacking of 2D metals on 2D semiconductors leads to the ideal Schottky junction or Ohmic contact by selecting 2D metallic materials with suitable work function. Liu et al. also employed graphene as contact electrodes and further encapsulated the MoS_2_ channel with top and bottom hexagonal boron nitride (h−BN). The MoS_2_ FETs reached a high field-effect mobility of up to 1300 cm^2^V^−1^s^−1^ at a low temperature [43]. Chuang et al. reported the use of Nb_0_._005_W_0_._995_Se_2_ as contact electrodes and the achievement of a low contact resistance (~0.3 kΩ·μm) [44]. Hwang et al. used chlorine-doped SnSe_2_ as the high-work-function contact metal in WSe_2_ FETs and realized a pronounced p-type Wse_2_ transistors with the mobility of 15.7 cm^2^V^−1^s^−1^, as shown in Figure 4c [45]. Wu et al. reported the fabrication of bi-layer WSe_2_ transistors via the vdW epitaxy and the controlled crack formation processes, as shown in Figure 4d [9]. In a 20 nm−long and 1.3 nm−thick bi-layer WSe_2_ transistor, an on-state current density of 1.72 mA μm^−1^ and a contact resistance of 0.25–0.54 kΩ·μm are achieved. PtSe_2_ has been demonstrated to have a higher electron mobility than MoS_2_ based on DFT calculations and experimentally extracted field-effect mobility [46]. Furthermore, PtSe_2_ shows a layer-dependent semiconductor to semimetal transition. When a PtSe_2_ transistor is built, few-layer PtSe_2_ can serve as a semiconducting channel and bulk PtSe_2_ can serve as the semi-metallic contact [47,48,49]. Das et al. vertically integrated a thick PtSe_2_ layer as source/drain contact on the surface of an ultrathin PtSe_2_ channel, achieving a high performance of all PtSe_2_ FETs, as shown in Figure 4e [50]. Zhang et al. reported barrier-free p-type WSe_2_ FETs with a layered 1T’−WS_2_ semimetal contact, as shown [51]. Owing to the high-quality interface between WSe_2_ and 1T’−WS_2_, the WSe_2_ FETs achieve a 50 meV Schottky barrier height and a high field-effect mobility of 97 cm^2^V^−1^s^−1^.

The growth of a graphene/MoS_2_ heterostructure and the use of graphene as contact have shown the potential to lower the contact resistance of MoS_2_ FETs [39]. In Mootheri et al.’s work, they further explored the function of 3D metal in the metal/graphene/MoS_2_ contact structure. They proved that Ru–graphene contact show the lowest contact resistance of 9.34 kΩ·μm compared with Pd–graphene and Ni–graphene contact [52].

The use of 2D metallic materials is a simple and effective way to achieve high-quality vdW contact on a 2D semiconductor. However, the stacking of 2D vdW heterostructures needs a complex transfer process during the device fabrication, which is inefficient for the fabrication of large-scale devices. Reliable transfer methods that are suitable for wafer-scale fabrication with a high alignment accuracy need to be explored. Furthermore, it is quite challenging to use the mechanical transfer method to fabricate short-channel devices. The etching of 2D layers with sub−1−micron precision is essential to realize the contact engineering of 2D FETs.

### 3.2. Edge Contact Engineering

Wang et al. first showed the structure of edge contact in 2D FETs by encapsulating a 2D channel with hexagonal boron nitride (h−BN) and exposing the edge of the channel to the metal contact. The edge contact shows several advantages, including being free of Fermi-level pinning induced by interfacial states and having a lower tunnel barrier, strong orbital overlaps, the absence of a Schottky barrier, and high carrier injection efficiency [53]. As the thickness of the 2D layer is very small, effective orbital overlap or hybridization is required between the metal and the edge of the 2D layers, which is the prerequisite to building high-quality edge contact. In monolayer TMDs such as MoS_2_ and WSe_2_, the CBM arises mainly from the d−orbitals of transition-metal atoms [54]. When a carrier is injected from metal to the conduction bands of MoS_2_, the edge contact exhibits strong orbital hybridization with transition-metal atoms [55] and leads to efficient carrier injection. In comparison, the top contact is formed on the surface of chalcogen atoms in monolayer MoS_2_ with little influence on the CBM. The realization of edge contact on 2D materials is mainly through plasma etching, metallization and phase engineering. We discuss these methods in this section.

#### 3.2.1. Plasma Etching and Metallization

**Figure 5:**
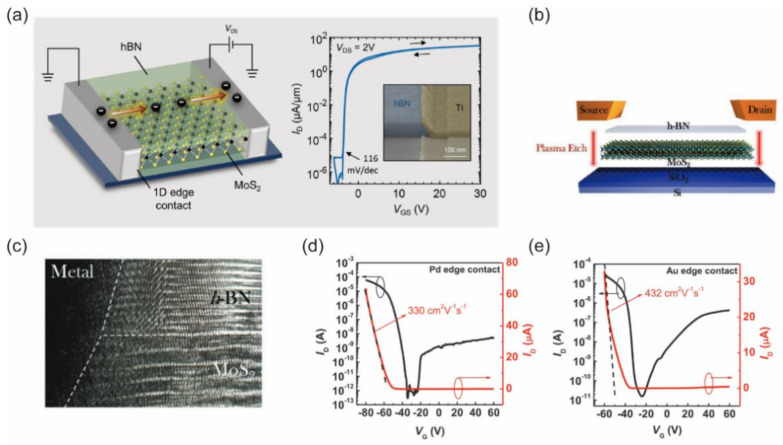
(**a**) Schematic of 1D edge contact MoS_2_ FET and transfer curve. Reprinted/adapted with permission from Ref. [56]. Copyright 2019, American Chemical Society. (**b**) Schematic diagram shows 1D edge contact FET with different metals. (**c**) HR−TEM image of Pd−MoS_2_ 1D edge contact FET; transfer curves of Pd (**d**) and Au (**e**) edge contact MoS_2_ FETs, realizing p-type intrinsic MoS_2_ FETs and Fermi-level depinning. Reprinted/adapted with permission from Ref. [55]. Copyright 2019, John Wiley and Sons.

Jain et al. reported edge-contact monolayer MoS_2_ FETs encapsulated with h−BN layers, as shown in Figure 5a [56]. The reactive ion etching, in situ Ar sputtering and annealing removed adsorbate on the MoS_2_ surface and preserved the high carrier mobility of ~30 cm^2^V^−^^1^s^−^^1^, resulting in a steep subthreshold swing of 116 mV/dec with a negligible hysteresis. Yang et al. reported the polarity control of MoS_2_ FETs by employing a 1D elemental metal contact (Figure 5b) [55]. Figure 5c shows the high-resolution transmission electron microscopy (HR−TEM) cross-sectional image of the edge contact area. The use of high-work-function palladium (Pd) or gold (Au) enables a high-quality p-type dominant contact to MoS_2_ layers without extrinsic doping, as shown in Figure 5d,e. Moreover, the h−BN encapsulation can suppress the interfacial scattering in 2D FETs and improve the long-term ambient stability, demonstrating the advantages of edge contact structure. Some 2D materials undergo gradual oxidation in air, especially MoTe_2_, black phosphorus and InSe, which can adopt the edge contact structure [57,58,59]. Except h−BN encapsulation, other insulating materials such as Al_2_O_3_ [60] and PMMA [61] have also been used to form edge contacts on 2D semiconductors.

#### 3.2.2. Phase Engineering and Degenerate Doping of 2D Layers

**Figure 6:**
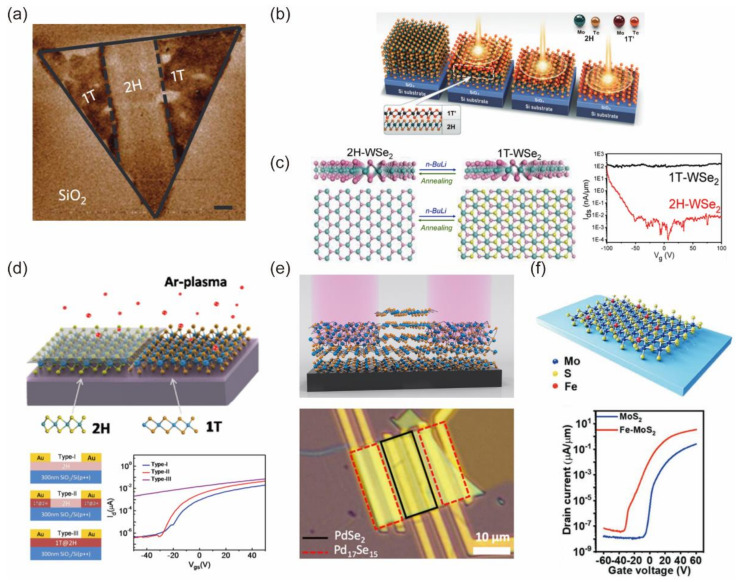
(**a**) Electrostatic force microscopy phase image of a monolayered MoS_2_ nanosheet showing the difference between locally patterned 2H and 1T phase. Reprinted/adapted with permission from Ref. [68]. Copyright 2014, Springer Nature. (**b**) Schematic showing laser-irradiation-induced phase transition from 2H to 1T’ Phase in MoTe_2_. Reprinted/adapted with permission from Ref. [70]. Copyright 2015, American Association for the Advancement of Science. (**c**) Side view and transfer curve comparison of the 2H and 1T phase WSe_2_. Reprinted/adapted with permission from Ref. [73]. Copyright 2015, American Chemical Society. (**d**) Schematic formation of 1T phase MoS_2_ via plasma treatment and three types of devices. Reprinted/adapted with permission from Ref. [74]. Copyright 2017, American Chemical Society. (**e**) Schematic and optical image of a PdSe_2_ device with irradiated contact area used to obtain the pristine channel and Pd_17_Se_15_ contacts. Reprinted/adapted with permission from Ref. [75]. Copyright 2019, American Chemical Society. (**f**) Controllable synthesis and doping determination of monolayer Fe−MoS_2_. Reprinted/adapted with permission from Ref. [77]. Copyright 2022, John Wiley and Sons.

Two-dimensional TMDs have been reported with different polymorphs, including hexagonal (2H) and monoclinic or octahedral (1T, 1T’) structures [62,63,64]. The 2H−phases MoS_2_ and WSe_2_ show semiconducting properties, while the 1T (1T’) phase displays metallic transport behavior. Therefore, phase engineering between 2H and 1T (1T’) can dramatically change the electronic properties of group−6 TMDs. The transition of group−6 TMDs from 2H to 1T (1T’) phase at the contact region can be used to achieve high-quality edge contact in 2D FETs, which is similar to the degenerate doping at the source/drain region [65,66,67]. Kappera et al. first demonstrated the phase transition of MoS_2_ from 2H to 1T through n-butyllithium treatment, as shown in Figure 6a [68]. The 1T/2H interface dominates the carrier injection, and the device exhibits an ultra-low contact resistance of 200–300 Ω·μm at zero gate bias. However, this 1T−phase MoS_2_ is metastable, and the stability is a challenge. This method can be used not only in MoS_2_ FETs [69], but also in other 2D-materials-based FETs. Cho et al. reported the laser-induced phase transition of MoTe_2_ from 2H to 1T’ phase, as shown in Figure 6b [70]. The 1T’ phase region works as the edge contact of the 2H phase channel to improve the carrier injection, and the Schottky barrier height is decreased to 10 meV. They further reported the reversible phase transition of MoTe_2_ between 2H and 1T’ by controlling the annealing temperature and the cooling speed [71]. The 1T’ MoTe_2_ has a thermal stability of 300 ℃, which is higher than 1T phase MoS_2_ [72]. Reversible phase transition of WSe_2_ layers has been reported by Ma et al. The n-butyllithium treatment on 2H-phase WSe_2_ induces the semiconducting to metallic phase transition, and the thermal annealing drives the metallic phase Wse_2_ to be converted back to the semiconducting phase, as shown in Figure 6c [73].

The generation of defects by weak plasma treatment can also Induce phase transition. Zhu et al. reported a facile, clean, controllable and scalable phase-engineering technique for monolayer MoS_2_, as shown in Figure 6d [74]. Point defects (single S vacancies) result in the 2H to 1T phase transitions. Akinola et al. also reported a phase transformation in a region of a layered semiconductor PdSe_2_, as shown in Figure 6e [75]. This phase transition is driven by defects created by argon plasma, and this turns PdSe_2_ into Pd_17_Se_15_. Recently, Cai et al. performed plasma treatment on patterned MoS_2_ layer to induce a local bonding distortion. This distorted area works as a semi-metallic bridge between the metal and the pristine channel to facilitate the charge injection [76]. The TEM image shows that the distorted MoS_2_ displays an octahedral structure, and the device exhibits an ultra-low contact resistance of 90 Ω·μm, approaching the quantum limit.

Another strategy is the introduction of degenerate doping during the growth process. Li et al. reported that unidirectionally aligned monolayer Fe−doped MoS_2_ domains are prepared on two-inch commercial c-plane sapphire, suggesting the feasibility of synthesizing wafer-scale-doped 2D semiconductors with outstanding device performance, as shown in Figure 6f [77]. Vu et al. reported a one-step growth approach to synthesize Nb-doped WSe_2_ with a controllable doping concentration. The fabricated NbSe_2_/doped-semiconductor vdW heterostructures have a low contact resistance of 2.46 kΩ·μm [78]. Hemanjaneyulu et al. reported the dramatic n-doping of MoS_2_ by immersing it in KI solution. The contact resistance can be greatly reduced to 0.75 kΩ µm [79]. Metallic nanoparticles have also been used to dope a 2D semiconductor channel and further effectively modulate the carrier transport in 2D FETs. Khan et al. reported the charge doping of ReSe_2_ through the adsorption of Co nanoparticles [80]. Sarkar et al. reported the doping effect of noble metal nanoparticles (Au, Ag, Pd, Pt) in TMDs and revealed the relationship between metal work function and the doping effect in MoS_2_ [81].

### 3.3. Inserting Interlayer between Metal and 2D Materials

**Figure 7:**
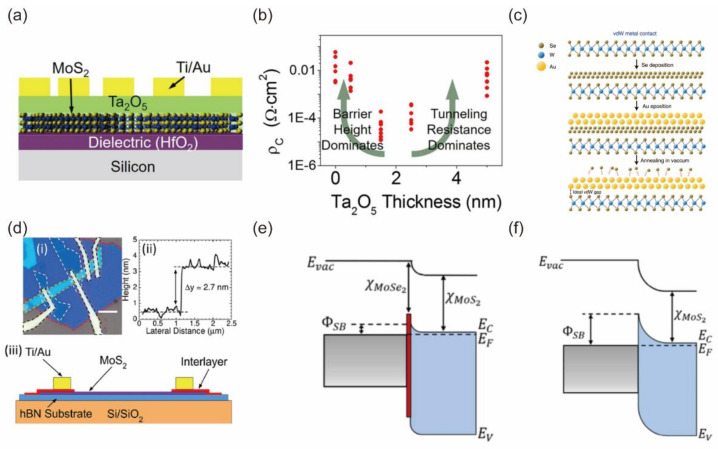
(**a**) Schematic of MoS_2_ FETs with various thicknesses of Ta_2_O_5_ interlayer. (**b**) Measured specific contact resistivity as a function of Ta_2_O_5_ dielectric thickness. Reprinted/adapted with permission from Ref. [84]. Copyright 2016, American Chemical Society. (**c**) Schematic of vdW Au contact WSe_2_ fabrication process. Reprinted/adapted with permission from Ref. [88]. Copyright 2022, Springer Nature. (**d**) (i) Optical micrograph of MoS_2_ FETs with MoSe_2_ interlayers. (ii) Thickness analysis of the MoSe_2_ interlayers. (iii) Device structure of MoS_2_ FETs with Ti/MoSe_2_ interlayer contacts. Illustrations of the band alignments at the contacts with a MoSe_2_ interlayer (**e**) and direct metal contacts (**f**). Reprinted/adapted with permission from Ref. [89]. Copyright 2020, American Chemical Society.

In previous sections, contact engineering has been realized by using 3D or 2D metallic materials, performing phase transition and introducing degenerate doping. Another strategy to suppress the Fermi-level pinning effect is introduced in this section. To decouple the orbital overlap, a thin insulating tunnel layer is inserted between the metal and semiconductor. The insulating buffer layer will increase the distance between the metal and semiconductor, which is an efficient way to suppress interface interaction, and the interlayer will inhibit high energy damage induced by the metal deposition process. The decrease in MIGS results in a reduced Schottky barrier height [82]. However, the thickness of the interlayer should be properly tuned because the electron tunneling through the insulating buffer layer is mandatory. Furthermore, the charges are injected by direct tunneling or Fowler–Nordheim tunneling dependent on the band alignment. Chen et al. first reported the insertion of a thin MgO film for Co−contacted monolayer MoS_2_ FETs [83]. The Schottky barrier height was reduced from 60 to 9.7 meV with the increasing MgO thickness from 0.9 to 2 nm. Lee et al. showed a statistical study of Schottky barrier height by inserting a thin tunneling Ta_2_O_5_ layer between MoS_2_ and metal contacts, as shown in Figure 7a [84]. They pointed out that a thin tunnel layer with a sub−2 nm thickness could allow efficient tunneling, as shown in Figure 7b. The remarkably suppressed Fermi-level pinning has also been demonstrated with other dielectric layers, such as h−BN [82,85,86], ZnO [87] and TiO_2_ [14]. Kwon et al. reported that defect-free vdW contacts were formed via a metal-deposition process with a selenium buffer layer on 2D layers, as shown in Figure 7c [88]. The device obeyed the Schottky-Mott rule and had a Fermi-level pinning factor of 0.91. Andrews et al. achieved a low Schottky barrier height of 25 meV by using MoSe_2_ as an interlayer between MoS_2_ channel and Ti electrodes, as shown in Figure 7d [89]. The reduction in Schottky barrier height can be attributed to the synergetic effect of Fermi-level pinning close to the conduction band edge of the MoSe_2_ interlayer and the favorable conduction band offset between the MoSe_2_ interlayer and MoS_2_ channel, as shown in Figure 7e,f.

## 4. Determination Methods of Contact Resistance

The determination method of contact resistance in 2D FETs should be consistent in different works for ease of comparison. There are three commonly used methods, including the transfer length method (TLM), Y−function method and four-point probe method.

### 4.1. Transfer Length Method

**Figure 8:**
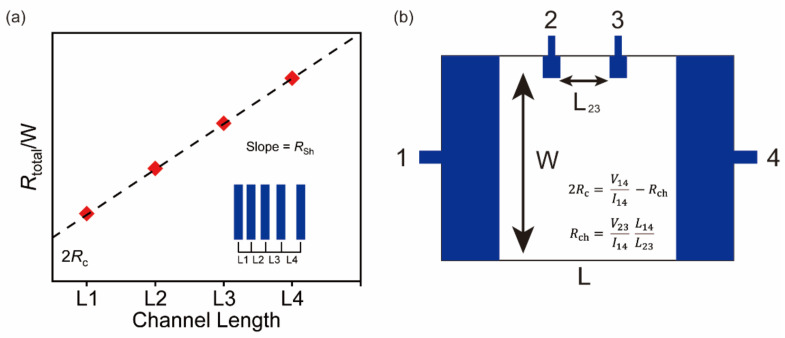
Methods to extract contact resistance. (**a**) Schematic of transfer length method. (**b**) Schematic of four-point probe method.

The transfer length method is widely used in 2D FETs to extract contact resistance [35]. The device should be fabricated with different channel lengths, as shown in Figure 8a. $R_{total}$ is the resistance between sourse and drain electrodes, $R_{sh}$ is the channel sheet resistance and W is the channel width. When $R_{total}/W$ is plotted versus the channel length, the *y*-axis intercept of the fitting line is equal to $2R_{c}$.

The contact resistance value extracted by TLM can have large variation when the channel length is large and the sheet resistance is huge. To minimize the estimated error, short-channel devices should be used, and statistic results are preferred.

### 4.2. Y-Function Method

The Y-function method requires only one transfer curve $I_{d}-I_{g}$ at the linear regime by applying a large gate voltage and a small source-drain voltage $V_{d}\ll V_{g}$ [90]. When the transconductance starts to decrease, the contact effect will dominate the μ attenuation and the contact resistance can be derived.

We assume that the contact resistance is comparable with the channel resistance. The source-drain voltage will drop at the contact region and $I_{d}$ can be expressed as the following equation

(4) $$I_{d}=\frac{\mu_{0}}{1+\theta_{0}\left(V_{g}-V_{th}\right)}C_{i}\frac{W}{L}\left(V_{g}-V_{th}-0.5V_{d}\right)\left(V_{d}-I_{d}R_{c}\right)$$

where $\mu_{0}$, $\theta_{0}$ and $V_{th}$ are the intrinsic mobility in the linear regime, first-order mobility attenuation coefficient, and the threshold voltage, respectively. When $V_{g}-V_{th}\gg 0.5V_{d}$, $0.5V_{d}$ can be ignored. The effective mobility attenuation factor θ represents the contribution from both $\theta_{0}$ and $R_{c}$. Therefore, $I_{d}$ can be written as the following equation

(5) $$I_{d}=\frac{\mu_{0}}{1+\theta \left(V_{g}-V_{th}\right)}C_{i}\frac{W}{L}\left(V_{g}-V_{th}\right)V_{d}$$

The Y—function was defined as

(6) $$Y=\frac{I_{d\;}}{\sqrt{g_{m}}}=\frac{I_{d\;}}{\sqrt{I_{d\;}/\left[1+\theta \left(V_{g}-V_{th}\right)\right]\left(V_{g}-V_{th}\right)}}=\sqrt{\mu_{0}C_{i}V_{d}\frac{W}{L}}\left(V_{g}-V_{th}\right)$$

where $g_{m}$ is transconductance $g_{m}=\partial I_{d}/\partial V_{g}$. The value $s_{1}$ can be extracted from the slope of the Y−function versus $V_{g}$. The value $s_{2}$ can be extracted from the slope of $\frac{1}{\sqrt{g_{m}}}$ versus $V_{g}$. The $R_{c}$ follows the equation:

(7) $$R_{c}=\frac{s_{2}}{s_{1}}V_{d}$$

### 4.3. Four-Point Probe Method

The four-point probe method to extract contact resistance requires the fabrication of a device with the desired structure, as shown in Figure 8b. The contact resistance is given by the following equation:

(8) $$2R_{c}=\frac{V_{14}}{I_{14}}-\frac{V_{23}}{I_{14}}\;\frac{L_{14}}{L_{23}}$$

## 5. Conclusions and Outlook

This review focused on the contact engineering of 2D FETs and discussed the origins of high contact resistance, the structure of top contact and edge contact, and the contact engineering in both structures. We believe that Fermi-level pinning in 2D devices is dominantly induced by interfacial gap states, and the solution to this challenge is to make a sharp and clean vdW interface at the contact regions. The top contact is compatible with the conventional Si CMOS process, but it is very challenging to control the deposition condition to achieve a vdW interface. The edge contact can be used in both top- and bottom—gate 2D FETs, but the accurate etching of 2D materials with little damage should be developed by using the dry etching technologies, such as reactive ion etching, plasma etching and inductively coupled plasma etching. Realization of the edge contact requires a much more complex fabrication process than that of the top contact. Although the edge contact methods can often achieve ultra-low contact resistance, the small contact area still limits the electrical performance of 2D FETs, such as on-state current. The insertion of a buffer layer provides another pathway to reduce the Fermi-level pinning effect, which can be combined with other contact-engineering strategies. In 2D GAAFETs, it is necessary to vertically integrate 2D FETs into integrated circuits, which is more challenging to achieve a good metal contact.

Overall, it is important to develop a CMOS-compatible contact deposition process to achieve large-scale 2D FETs with high-performance transport properties. One promising method is to employ an alloy composed of low-melting-point metal and high-melting point-metal as contact to simultaneously achieve vdW contact and increase the temperature endurance for the BEOL process. Another promising method is to build a high-quality mixed contact by combining the advantages of edge contact and top contact to overcome the small contact areas and Fermi-level pinning.

## Data Availability

Not applicable.
